# Frequency and Pattern of Worldwide Ocular Gene Therapy Clinical Trials up to 2022

**DOI:** 10.3390/biomedicines11123124

**Published:** 2023-11-23

**Authors:** Hossein Ameri, Niranjana Kesavamoorthy, Dara N. Bruce

**Affiliations:** Department of Ophthalmology, USC Roski Eye Institute, Keck School of Medicine, University of Southern California, Los Angeles, CA 90033, USA; kniranjana828@gmail.com (N.K.);

**Keywords:** ophthalmic, ocular, gene therapy, clinical trials, gene augmentation, viral vectors, inherited retinal diseases, retinitis pigmentosa, Leber congenital amaurosis, age-related macular degeneration

## Abstract

The purpose of this study is to describe worldwide gene therapy clinical trials aimed at treating ophthalmic disorders. Information regarding all worldwide clinical trials was collected through 15 different sources, including ClinicalTrials.gov. There were 159 gene therapy clinical trials on ophthalmic diseases up until 2022. Phase 1/2 trials had the highest frequency (50—32%), followed by phase 2 (33—21%); 107 trials (67%) were conducted in a single country, and 50 trials (31%) were multinational. Overall, the USA was the site of 113 (71%) single or multinational trials. Of the trials, 153 (96%) targeted retina and optic nerve disorders, 3 (2%) glaucoma, 2 (1%) uveitis, and 1 (1%) cornea; 104 trials (65%) employed gene augmentation using viral vectors, and the remaining employed other methods such as inhibitory RNA (18—11%) and cell-based gene therapy using encapsulated cell technology (18—11%). For gene augmentation trials, adeno-associated virus was used for transgene delivery in 87% of cases. The most common conditions targeted by gene augmentation included inherited retinal (74%) and age-related macular degeneration (wet, 14%; dry, 7%). Overall, a large number of gene therapy clinical trials have been conducted in the eye, and so far, one has led to regulatory approval.

## 1. Introduction

Since the 1950s, when the DNA structure was discovered, gene modification has been entertained as a means of changing a human or an organ for a specific purpose. Further understanding of human biology and the discovery of some genes, as well as the development of biological vehicles for gene delivery, resulted in the introduction of the first gene therapy clinical trials in 1990 [1,2]. More than a decade later, the first gene therapy was approved for cancer treatment in 2003 [3]. In 2017, the FDA approved Voretigene neparvovec-rzyl for RPE65-mediated retinal dystrophy. It was the first FDA-approved gene therapy treatment for any inherited disease [4]. That approval was the result of two decades of research and a decade of human clinical trials. Despite vision scientists and ophthalmologists being at the forefront of gene therapy efforts, studies have severely undercounted ocular gene therapy clinical trials [5,6]. The purpose of this study was to provide an overall description of worldwide gene therapy clinical trials targeting ocular diseases.

## 2. Materials and Methods

The ocular gene therapy clinical trials listed in our paper have been compiled by obtaining information from various online sources including US National Institute of Health ClinicalTrials.gov [7], Wiley database on Gene Therapy Trials Worldwide [8], International Standard Randomised Controlled Trial Number Register [9], Gene Therapy Net [10], EU Clinical Trials Register [11], Australia New Zealand Clinical Trials Registry (ANZCTR) [12], EU Deliberate release database on clinical trials (JRC) [13], Chinese Clinical Trial Registry (ChiCTR) [14], US National Cancer Institute Cancer Trials [15], Belgian Biosafety Server Clinical Trials Database [16], World Health Organization International Clinical Trials Registry Platform [17], UK Gene Therapy Research [18], German Clinical Trials Register [19], and Japan Registry of Clinical Trials (JRCT) [20], UMIN Clinical Trials Registry (UMIN-CTR) [21].

The search was performed using various keywords, which were determined based on our prior knowledge of past or present clinical trials through publications, meeting presentations, and press releases. These often resulted in finding specific trials. To capture all trials, additional searches were performed using the names of diseases or genes. The trials were then screened, and those involving gene therapy were selected. ClinicalTrials.gov was by far the most comprehensive registry, with 86% of trials listed. Seventy-two trials were found to be listed in more than one registry; the duplicates were deleted.

## 3. Results

**Figure 1:**
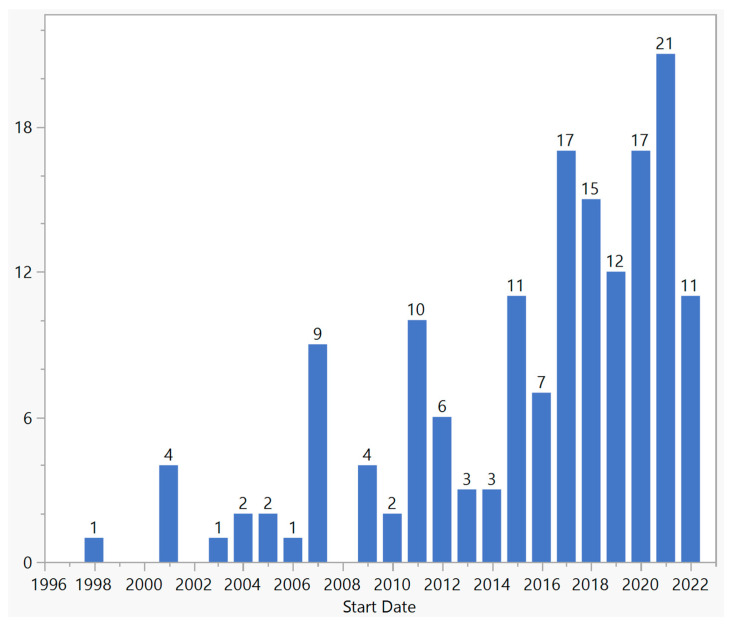
Distribution of ocular gene therapy clinical trials by start year.

There were a total of 159 gene therapy clinical trials targeting ophthalmic disorders up until the end of 2022 (Figure 1). Approximately 7130 patients were enrolled in these trials, and the number of participants per trial ranged from 1 to 465. The first trial, which targeted gyrate atrophy, was sponsored by the National Eye Institute and started in 1998. This was in fact a systemic ex vivo gene therapy treatment in which skin keratinocytes were biopsied, grown in the laboratory, inserted with a functional copy of the ornithine aminotransferase gene, and grafted back into the patient’s skin. The study was completed in 2000. In all other trials, the treatments were performed on the eye. Since 2015, there has been a considerable growth in the number of new trials, with a total of 111 trials and an average of 14 trials per year. The highest number of new trials was in 2021, in which 21 trials were started. Among all the trials, one trial (0.6%) led to an FDA-approved product.

**Figure 2:**
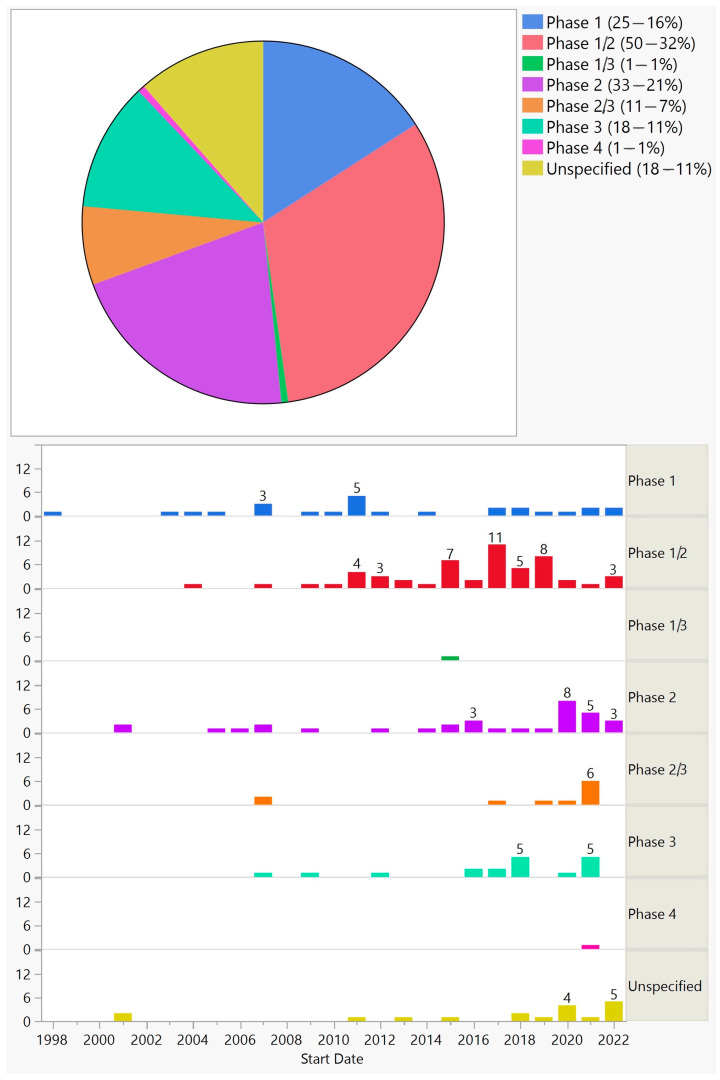
Phases of ocular gene therapy clinical trials (**top**) and their distribution by start year (**bottom**).

Overall, the frequency of trials for phases 1, 1/2, 2, 2/3, 1/3, 3, and 4 was 25 (16%), 50 (32%), 33 (21%), 11 (7%), 1 (1%), 18 (11%), and 1 (1%), respectively; 18 (11%) did not specify the phase (Figure 2).

**Figure 3:**
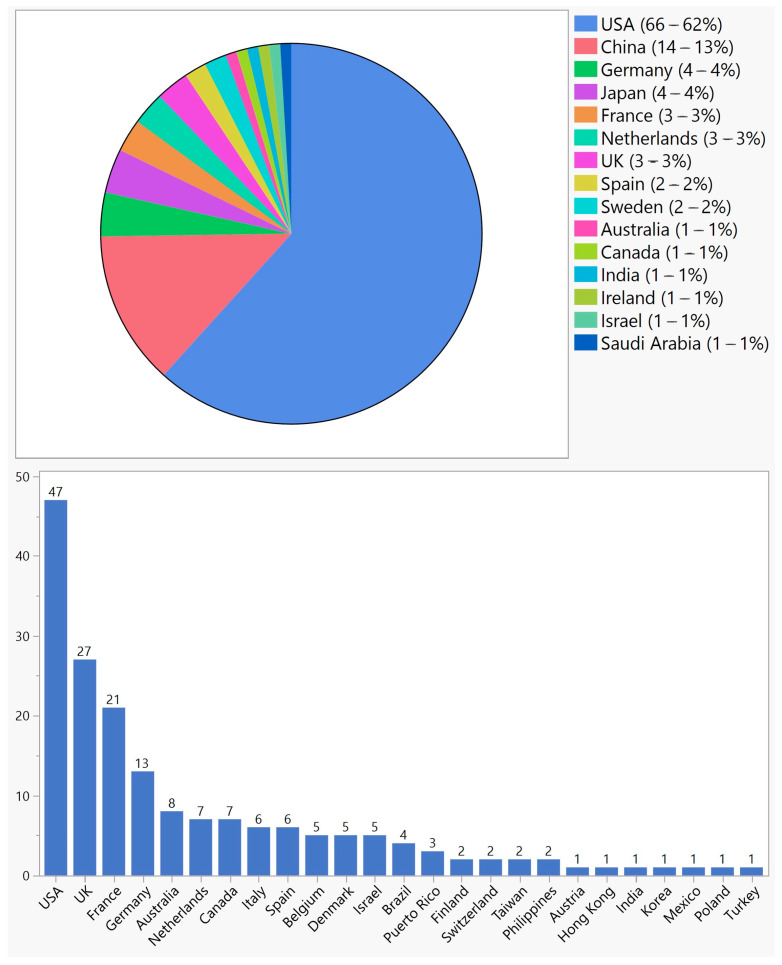
Geographical distribution of ocular gene therapy clinical trials in single-country trials (**top**) and multinational trials (**bottom**).

107 (67%) trials were conducted in a single country, with the USA having the highest share (Figure 3). However, 50 (31%) trials were multinational, 47 (94%) of which were conducted in the USA and one or more countries (Figure 3). Overall, the USA was the site of 113 (71%) single or multinational trials. Twenty-nine (58%) multinational trials were in two countries, five (10%) in three countries, two (4%) in four countries, and fourteen (28%) in five countries or more.

The majority of trials (118—74%) were sponsored by industry, and the remaining (41–26%) were sponsored by academic centers. While 56 (35%) were randomized, 100 (63%) were open-label. Seventy (44%) trials were completed, and eight (5%) were prematurely terminated or withdrawn for various reasons; often business-related decisions were stated as the cause, and none has reported safety reasons for early termination. Further, 153 (96%) trials targeted retina and optic nerve disorders, 3 (2%) glaucoma, 2 (1%) uveitis, and 1 (1%) cornea.

**Figure 4:**
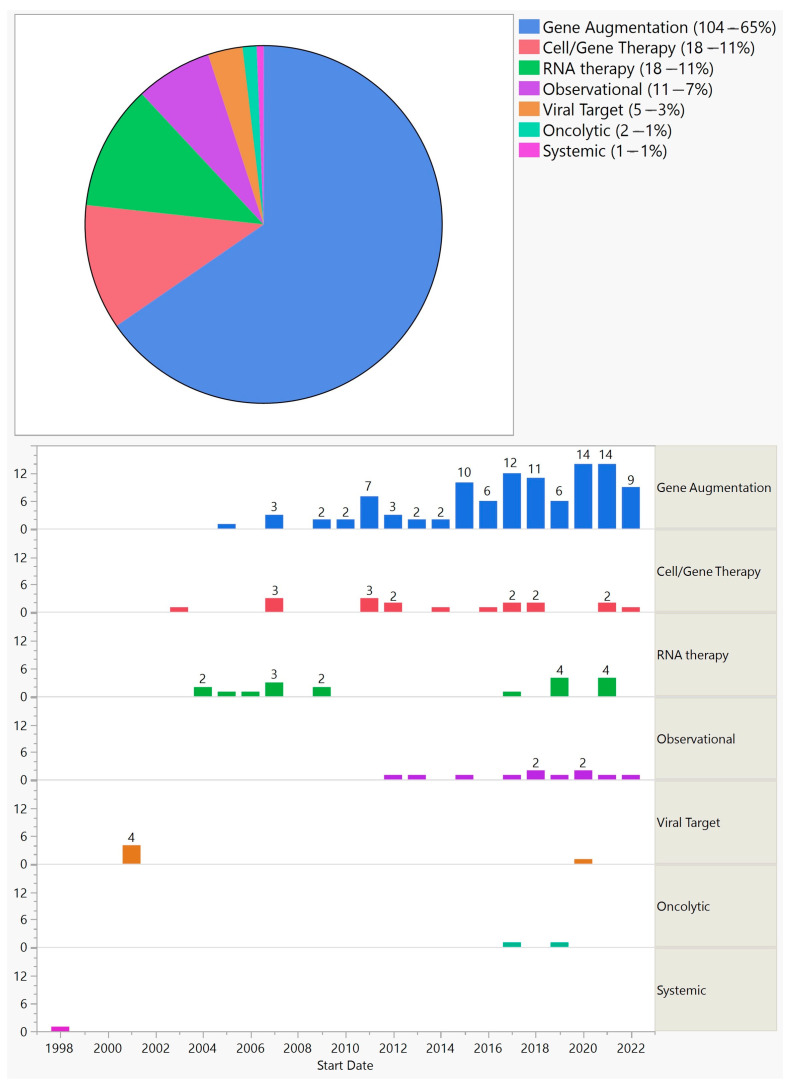
Categories of ocular gene therapy clinical trials (**top**) and their distribution by start year (**bottom**).

The trials could be divided into several categories: gene augmentation (104—65%), cell-based gene therapy–encapsulated cell technology (18—11%), RNA therapy (18—11%), gene therapy targeting viruses (5—3%), oncolytic (2—1%), and observational extension of gene augmentation therapies (11—7%) (Figure 4).

### 3.1. Gene Augmentation

**Figure 5:**
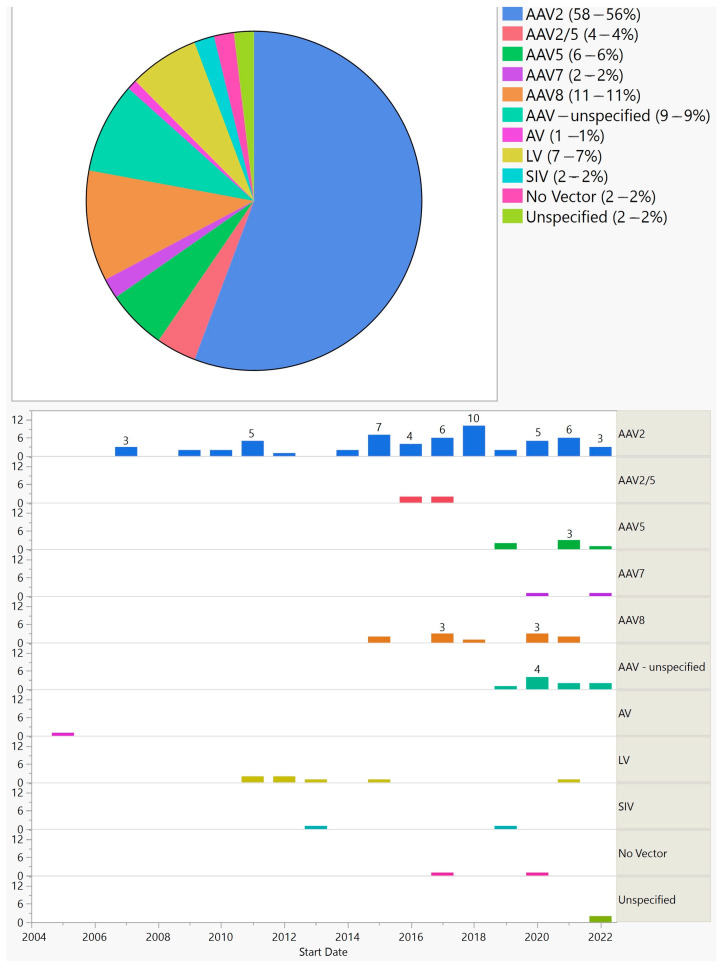
Vectors used in ocular gene augmentation trials (**top**) and their distribution by start year (**bottom**).

Apart from early trials that targeted viruses or used RNA therapy, gene augmentation has been the predominant method and has shown steady growth over the years. Similar to all trials, phase 1/2 had the highest frequency. With few exceptions, all gene augmentation trials used adeno-associated virus (AAV), albeit of different serotypes (Figure 5).

Furthermore, 90 (87%) trials used AAV, 7 (7%) lentivirus, 2 (2%) simian immunodeficiency virus, and 1 (1%) adenovirus. Two (2%) that aimed at treating uveitis delivered DNA plasmid using electrotransfer instead of a viral vector.

**Figure 6:**
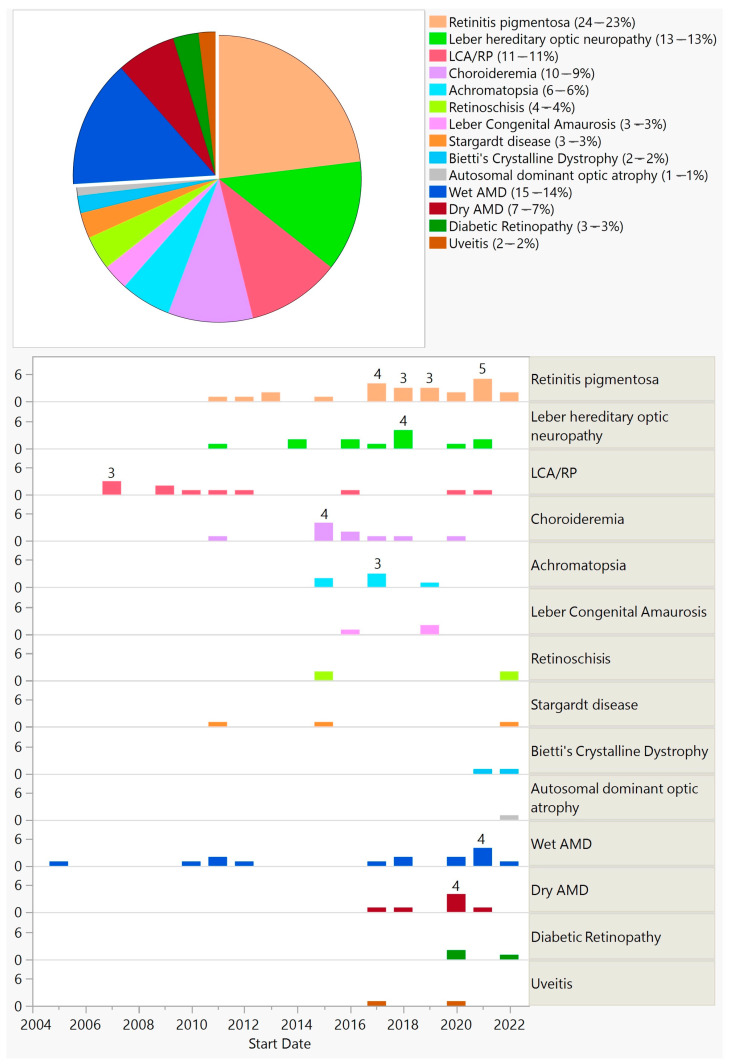
Diseases targeted in ocular gene augmentation trials (**top**) and their distribution by start year (**bottom**). The separated piece of pie indicates acquired conditions (**top**).

Gene augmentation was employed to treat both inherited and acquired conditions. (Figure 6).

Furthermore, 77 (74%) trials aimed at treating inherited diseases by targeting a specific gene, with the exception of 9 trials that were gene-agnostic and introduced channelrhodopsin genes (optogenetics—7 trials) or the hPEDF gene (2 trials) (Figure 6). Twenty-seven (26%) trials that aimed at treating acquired conditions introduced genes targeting various pathways. One trial used CRISPR-Cas9 technology delivered via AAV5 for treating CEP290-mediated Leber congenital amaurosis (LCA).

Gene augmentation treatment was delivered via subretinal injection in 59 (57%), intravitreal injection in 38 (37%), suprachoroidal injection in 3 (3%), and topical with electrotransfer in 2 (2%).

### 3.2. Cell-Based Gene Therapy

There were 18 trials that used encapsulated cell technology. In all of these trials, immortalized retinal epithelial cells were genetically modified to produce ciliary neurotrophic factor (CNTF) in a polymer membrane capsule implanted into the vitreous cavity. The first trial of this kind started in 2003. Twelve trials targeted acquired conditions (macular telangiectasia—seven trials; glaucoma—three trials; ischemic optic neuropathy—one trial; atrophic macular degeneration—one trial) and six trials targeted inherited diseases (retinitis pigmentosa (RP)—five trials; achromatopsia—one trial). There were three phase 3 trials on macular telangiectasia and two phase 2/3 trials on RP. Fourteen trials completed their studies.

### 3.3. RNA-Based Therapy

There were 18 trials that used RNA technology. The first trial of this kind started in 2004. Unlike other gene therapy methods, RNA therapy is short-acting and often requires repeated injections. All treatments were delivered via intravitreal injections. Half of the trials (nine trials) targeted wet age-related macular degeneration (AMD), and the other half (nine trials) targeted inherited diseases (RP—five trials; LCA—four trials). Five trials were withdrawn, and nine trials have completed their studies. There were two phase 3 trials on treating wet AMD, both of which were withdrawn. There were five phase 2/3 trials: three trials targeting RP and two trials targeting LCA; one trial targeting RP was withdrawn.

### 3.4. Miscellaneous

There were 11 observational gene therapy trials in which no interventions were performed, but rather patients from previous interventional trials were enrolled for long-term follow-up. Eight trials were on inherited diseases (RP—five trials; Usher syndrome—one trial; Stargardt disease—one trial; Achromatopsia—trial) and three on acquired conditions (wet AMD—two trials; dry AMD—one trial). Nine trials were still ongoing at the end of 2022.

There were five gene therapy trials that directly targeted viruses. Four of these trials, which targeted cytomegalovirus, were delivered through intravitreal injections, and all started in 2001 in the US. One trial started in 2020 in China, using CRISPR-Cas9 technology to target viral keratitis caused by the herpes simplex virus. The treatment was delivered through an intracorneal injection.

Two trials used oncolytic viruses to treat ocular tumors; one trial started in Spain in 2017 and used oncolytic adenovirus for the treatment of refractory retinoblastoma. Another trial started in the USA in 2019 and used oncolytic vesicular stomatitis virus for the treatment of Stage III-IV melanoma, including metastatic choroidal melanoma. Two trials used CRISPR-Cas9: one for CEP290 and another for viral keratitis.

## 4. Discussion

Ocular gene therapy has emerged as a promising approach for the treatment of various ocular diseases. In this study, we aimed to provide an overview of ocular gene therapy clinical trials, their characteristics, and the different approaches employed in these trials. The field of ocular gene therapy started about a decade after the first human gene therapy clinical trial for systemic diseases. With 159 trials from 1998 to 2022, vision scientists and ophthalmologists have demonstrated a strong desire to develop novel treatments using various gene therapy approaches. In this period, a quarter of a century, only 4 years were without any new ocular gene therapy trials: 1999, 2000, 2002, and 2008. Over the years, there have been fluctuations in the number of new trials, but since 2015, there has been significant growth, reaching a maximum of 21 trials in 2021. Most trials were in early phases, but there were 18 phase 3 trials across various approaches. Notably, only one treatment resulted in an FDA-approved product, highlighting the challenging journey from clinical trials to regulatory approval. Although this success rate of 0.6% (number of approved products/number of trials) is comparable to other gene therapy products, it is 20 times less than the average success rate of 12%; based on a 2021 report by the Congressional Budget Office, 10–14 percent of drugs entering clinical trials ultimately receive FDA approval [22]. With 71% of trials conducted in the USA, this country played a major role in both single-country and multinational trials; 61% of single-country trials and 87% of multinational trials were conducted in the USA. However, other countries showed a different pattern of participation depending on whether the trial was single-country or multinational. For example, China and Japan, which have second and third places in single-country trials by 13% and 4%, respectively, were not part of any multinational trials. On the other hand, the UK, France, and Germany, which comprised 3%, 3%, and 4% of single-country trials, respectively, participated in 50%, 39%, and 24% of multinational trials, respectively.

Of the methods employed, gene augmentation has been the most common, with 104 trials between 2005 and 2022. This is important because gene augmentation has the potential to cure or permanently change the course of a disease. About three-quarters of gene augmentation trials targeted inherited diseases, and they were limited to retinal and optic nerve disorders. Out of these, 88% aimed at replacing a defective gene, and therefore, a product of such would be limited to a small group of patients. They would also be beneficial in the earlier stages of the disease, before extensive cell loss [4]. On the other hand, a small group of gene-augmentation trials were gene-agnostic and would be applicable to a much larger patient population. For example, optogenetic trials that were conducted on RP and Stargardt patients could potentially be used for any outer retinal disease as long as there is preservation of the inner retina. Another advantage of gene-agonistic approaches is that they could be beneficial in the advanced stages of the disease. However, the maximum resolution vision they could offer remains uncertain. Of the seven optogenetic trials, none were phase 3, meaning that we are at least a few years away from a regulatory-approved treatment of this kind. A quarter of gene augmentation trials targeted acquired conditions, predominantly wet and dry AMD, with a few trials on diabetic retinopathy and uveitis. Of the 27 gene augmentation trials for acquired conditions, all were early stage trials except one phase 2/3 and one phase 3 trial starting in 2020 and 2021, respectively; both trials are on wet AMD and are still ongoing. Two early gene-augmentation trials were withdrawn.

With the exception of two trials for uveitis that used electrotransfer for gene delivery, all gene augmentation trials utilized viral vectors. The first viral vector used in gene augmentation trials was adenovirus in 2005. No other trials used this vector afterward. With 87%, AAV is the most commonly used vector. AAV2 is by far the most commonly used AAV serotype; its use was started in 2007, and until 2013 it was the only AAV serotype used. In recent years, AAV 2/5, AAV5, AAV7, and AAV8 have been used in some trials; between 2019 and 2022, their cumulative use in each year was higher than that of AAV2. Lentivirus is another vector and was used in several trials between 2011 and 2015, and one trial in 2021. The delivery method for AAV has been intravitreal or subretinal, with the exception of two suprachoroidal injections of AAV8 (one for RP and another one for wet AMD; the lentivirus was delivered via subretinal injection, with the exception of one suprachoroidal delivery for wet AMD).

In 11% of ocular gene therapy trials, gene modification was performed ex vivo on immortalized retinal pigment epithelial cells, which were then implanted into the eye using encapsulated cell technology. The first of these trials started in 2003, two years before the gene augmentation trials, and since then there have been new trials, on and off, until 2022. As the implanted capsule produces CNTF, a growth factor, it is a nonspecific treatment and has therefore been used for both acquired and hereditary conditions. Macular telangiectasia, followed by RP, have been the most common targets of these trials. Three phase 3 trials on macular telangiectasia started in 2017 and 2018, but none have led to a regulatory-approved treatment. There have been no withdrawals of any trials in this category.

A total of 11% of ocular gene therapy trials used RNA-based therapy. By nature, this approach would not produce a permanent or long-term treatment. The first RNA therapy trials started in 2004. There was a gap in new trials between 2009 and 2017. Half of these trials targeted inherited diseases, and the other half targeted acquired conditions. Five (28%) of these trials have been withdrawn, which is much higher than any other category. The only two phase 3 trials, which started in 2007 and 2009, are among the withdrawn trials.

Limitations of this study include challenges in data collection. There is no single data registry that includes all trials. Therefore, despite our best efforts and extensive search, there might be a trial or trials that were not captured in our study. Another challenge is that registries vary in the amount of information provided for each trial. Moreover, in each registry, some trials do not provide a complete set of information. Fortunately, this was not a major problem, and throughout the paper, we stated whenever the information was not available for each category. Creation of a global registry with detailed information could facilitate future studies.

## 5. Conclusions

This study provides a comprehensive overview of ocular gene therapy clinical trials, highlighting the diverse approaches, vector types, and target diseases investigated in this field. Gene augmentation was the predominant method employed in these trials. Although the number of FDA-approved ocular gene therapies remains limited, the progress made thus far demonstrates the potential of gene therapy to revolutionize the treatment of ocular diseases. Continued research, collaboration, and regulatory support are crucial to further advance ocular gene therapy and translate promising interventions into effective and accessible treatments for patients with ocular disorders.

## Data Availability

The data presented in this study is contained within the article.
